# Re-irradiation in lung disease by SBRT: a retrospective, single institutional study

**DOI:** 10.1186/s13014-018-1041-y

**Published:** 2018-05-08

**Authors:** Donatella Caivano, Maurizio Valeriani, Sara De Matteis, Paolo Bonome, Ivana Russo, Vitaliana De Sanctis, Giuseppe Minniti, Mattia Falchetto Osti

**Affiliations:** 1 Departement of Radiation Oncology, CNAO (National Centre of Oncological Hadrontherapy), Str. Campeggi, 53, 27100 Pavia, Italy; 2 Department of Radiation Oncology, Faculty of “Medicina e Psicologia”, University of Rome “Sapienza”, Sant’Andrea Hospital, Via di Grottarossa 1035/1039, 00189 Rome, Italy; 3 UPMC San Pietro FBF, Centro di Radioterapia ad alta specializzazione, Via Cassia 600, 00189 Roma, Italia; 4 Neuromed - Mediterranean Neurological Institute, Via Atinense, 18, 86077 Pozzilli, (IS) Italy

**Keywords:** Re-irradiation, SBRT, Lung, Primary or metastatic disease

## Abstract

**Background:**

The loco regional relapse is frequent in the lung disease. The aim of this study was to evaluate the outcomes of re-irradiation by SBRT in terms of Local Control (LC) and toxicities.

**Methods:**

From April 2011 to December 2016, twenty-two patients received a re-irradiation by SBRT. Twenty- seven lesions were treated. The medium BED(10) of re-irradiation was 100.6 Gy (range: 48–151.2 Gy) and the medium EQD2(10) was 93.8 Gy (range: 40–126 Gy). In the previous treatment the medium BED(10) was 97.2 Gy (range: 40–120 Gy), the medium EQD2(10) was 81 Gy (range: 32.5–100 Gy). The median time between the first and the second treatment was 18 months.

**Results:**

Local Control was reached in 18 out of 27 (66%) re-irradiated lesions, with rates of 67 and 54% at 1- year and 2- years respectively. The treatment was well tolerated; the maximum recorded toxicity was Grade 3.

**Conclusions:**

Re- irradiation by SBRT may represent an option for the treatment of lung disease with good results in terms of LC and toxicity.

## Background

Lung cancer is one of the most important causes of death for cancer in the world. Patients with early-stage lung cancer have a probability of 4–10% developing a second lung tumor in the first 5 years after treatment [1, 2]. Patients with stage III Non-Small Cell Lung Cancer (NSCLC) treated with concurrent chemo-radiotherapy (CRT) have a risk of 30% for isolated locoregional failure [3]. The lungs are a common site for metastases. A considerable percentage of patients have disease limited to the thorax, which represents an ideal subset for local therapy [4]. A considerable number of patients with primary or metastatic lung lesions need to repeat thorax treatment for recurrent or metachronous disease. For this reason, thoracic re-irradiation starts to be common in clinical practice. Thorax recurrences can be separated in local (lung parenchyma, bronchial stump, or chest wall) and regional (mediastinal lymph nodes). The aim of treatment can be palliative or curative [5]. The improvements of technologies with Stereotactic Body Radiation Therapy (SBRT) have introduced the concept of ablative radiotherapy with curative intent. The SBRT is able to give a high dose of radiation sparing the surrounding normal tissue in 1–5 fractions [6]. The purpose of this study was to evaluate clinical outcomes in terms of Local Control (LC) and toxicity in patients with lung disease (primary or metastatic) which have received re-irradiation by SBRT. Also we have correlated Local Control with tumor histology, status of patient, number of fraction, doses, chemotherapy after treatment, time from the first progression of disease after the first treatment, time between both treatments. Furthermore, we have correlated toxicity with site of treatment, doses and volume.

## Methods

From April 2011 to December 2016, twenty-two patients received a re-irradiation by SBRT. Twenty-seven lesions were re-irradiated. There were 15 (68%) male and 7 (32%) female. Median age at time of enrolment was 70 years (range: 47–82 years). This study included patients with comorbidity or advanced age or patients with low cardio-respiratory reserve which were unsuitable for surgery or patients refusing surgery. The patients at time of re-irradiation had an Eastern Cooperative Oncology Group (ECOG), PS 0–2, and the intent of treatment was non-palliative. Patients and tumor characteristics are summarized in Table 1. The median time to the progression disease from first treatment was 10 months (range: 0–54 months). The median time between the first and the second treatment was 18 months (range: 6–66 months). For each patient we obtained the disease history and an objective examination. According to these informations we classified for ECOG, PS. The imaging study was performed using TBCT with contrast enhancement or with18FDG PET-CT to assess the status of systemic disease. The median follow-up time was 13 months (CI: 95%, range = 13–65 months).

**Table 1:** Characteristics of Patients

	Number	Percent
Total number of patients	22	
Males	15	68
Females	7	32
Primitive lung cancer	13	59
NSCLC	12	54
SCLC	1	4
PET Positive, No histological report	2	9
METASTASIS	7	32
Colon Cancer	2	10
Rectal Cancer	2	10
Breast Cancer	1	4
Carcinoma of the Uterus	1	4
Oropharyngeal Cancer	1	4
Histology		
Adenocacinoma	13	59
Squamous Carcinoma	6	27
SCLC	1	5
PET Positive, No histological report	2	9
Performace status		
0	12	55
1	9	41
2	1	4
Status of patients		
Patients with only one site of disease	4	15
Patients with others site of disease, but stable	23	85
Chemotherapy after re-irradiation		
Yes	8	30
No	19	70
Diagnosis of recurrence disease		
PET-CT 18FDG	19	70
TBCT	8	30
Histological Exam	0	0
Localization of lesions		
Peripheral	23	85
Central	4	15

### Treatment plan

All patients underwent computed tomography (CT) simulation and were immobilized in the supine position. A 4DCT system was used to generate an Internal Target Volume (ITV) to evaluate respiratory excursion. The ITV was created on the MIP (Maximum Intensity Projection). When the differentiation between post-surgical/post-radiotherapy scarring and recurrent tumor was not well visible, a fusion of images with 18 FDG PET-CT was done to better define it. The sum of GTV (Gross Tumor Volume) and ITV was expanded by 4 mm in all directions to create the PTV (Planning Tumor Volume). The SBRT plan was created with a conformational 3D technique, using multiple fields (5–7 fields), non-opposing and coplanar. The energy was 6–15 MV. The median prescription dose was 98% (range: 94–99%). The characteristics of lesions are summarized in Table 2. We considered re-irradiation in-field when there was overlap of the two separate dose distributions for the 80% dose level each time [7].

**Table 2:** Characteristics of the Lesions

	Number	Range
Total number of lesions	27	
Volume		
Medium Volume (cc)	30,8	2,7–260,7
Diameter		
Medium Diameter (cm)	3,7	1,7–7,9
Localization of lesions		
Out-field	6	22
In-field	21	78

### Doses

Seven lesions (26%) were re-irradiated with a single fraction and twenty (74%) lesions were treated with multiple fractions. Table 3 summarized the doses and the number of fractions of first treatment and re-irradiation. At the time of re-irradiation, the choice of single fraction vs. multiple fractions was based on the volume of lesions and on the proximity of the target to critical organs, such as large vessels or bronchial shaft. The doses were measured also in terms of BED (Biological Effective Dose) and EQD2 (Equivalent Dose in 2 Gy), assuming that the α/β ratio is 10 for tumor and acute toxicity [8]. The doses in terms of BED (10) and EQD2 (10) are summarized in Table 3. For each treatment there was the DVH (Dose Volume Histogram) that evaluated the dose received by the organs at risks and by the target. It was not possible to sum the DVH of the first treatment and second treatment because there was not algorithm or image system that permitted it.

**Table 3:** Number of Fractions and Doses

Number of fractions of the first treatment (SBRT)	Number	Percent
Single Fraction		
30 Gy/ 1 fr	11	42
23 Gy/1 fr	3	11
Multiple Fractions		
45 Gy/ 3fr	3	11
50 Gy/5 fr	3	11
60 Gy/ 30 fr ^**a**^	5	18
30 Gy/ 10 fr ^**b**^	2	7
Number of fractions of the re-irradiation (SBRT)		
Single Fraction		
30 Gy/ 1 fr	4	15
23 Gy/1 fr	3	11
Multiple Fractions		
54 Gy/ 3 fr	3	11
45 Gy / 3fr	5	18
50 Gy/ 5fr	8	30
30Gy/fr 5	4	15
Medium biological effective dose (10Gy) (BED10)	Dose	Range
I Treatment	97,2 Gy	40–120
Re-Irradiation	100,6 Gy	48–151,2
Medium equivalent dose in 2 Gy (10 Gy) (EQD2)	DOSES	RANGE
I treatment	81 Gy	32,5–100 Gy
Re-Irradiation	93,8 Gy	40–126 Gy
Cumulative medium BED (10)	197,8 Gy	88–271,2 Gy
Cumulative medium EQD2 (10)	164,8 Gy	72,5–226 Gy
Median biological effective dose (10Gy) (BED10)	Doses	Range
I Treatment	112,5 Gy	40–120 Gy
Re-Irradiation	100 Gy	48–151,2 Gy
Median equivalent dose in 2 Gy (10 Gy) (EQD2)	Doses	Range
I treatment	93,8 Gy	32,5–100 Gy
Re-Irradiation	83,3 Gy	40–126 Gy
Cumulative median bed (10)	200 Gy	88–271,2 Gy
Cumulative median EQD2 (10)	166,7 Gy	72,5.226 Gy

^a^Treatment done by IMRT technique

^b^ Treatment done by 3D technique

### Follow up

The first follow-up was at 2 months. We evaluated physical examinations, blood tests, tumor markers and TBCT with contrast enhancement. Then every 3 months for 2 years by first follow-up, after that every 6 months. The first imaging was done by CT scan with contrast enhancement, and then patients had alternatively 18 FDG PET-CT and TBCT. The criteria for radiological response evaluation were extrapolated from the RECIST 1.1 scale (Solid Tumors Response Evaluation Criteria). Toxicity was classified by the CTCAE scale v.4.

### Statistical analysis

The first end point was the Local Control (LC) and the second end point was toxicity. The LC is defined as the absence of new or progressive lesions within or at the margin of the PTV re-irradiated. In the statistical analysis we described also the Progression Free Survival (PFS), the Disease Specific Survival (DSS) and the Overall Survival (OS). The PFS was calculated from the first day of SBRT (re-irradiation) and was defined absence of any local and distant disease. The DSS was calculated from the first day of treatment (re-irradiation) until death for disease. Patients died for causes not related to disease were not considered as event. The OS was calculated from the first day of re-irradiation by SBRT until death. LC, PFS, DSS and OS were estimated using the Kaplan-Meier method. Differences between survival curves and tumor histology, status of patient, number of fraction, doses, chemotherapy after treatment, time from the first progression of disease after the first treatment and time between both treatments were tested with log-rank test analysis. Statistical analyses were performed using the software package SPSS, version 24 (IBM corporation). A *p* ≤ 0.05 was considered statistical significant.

## Results

The results in terms of Local Control and survival index are showed in Table 4, those of toxicity in Table 5. Finally, the correlation between the LC, survival index and variables are showed in Table 6.

**Table 4:** Index of survival and Local Control

	1 Year	2 Years
^a^LC	67%	54%
^b^PFS	45%	39%
^c^DSS	81%	63%
^d^OS	81%	63%

^a^Local Control

^b^Progression Free Survival

^c^Disease Specific Survival

^d^Overall Survival

**Table 5:** Toxicity

		^a^Acute			^b^Late			
Grade	1	2	3	4	1	2	3	4
Dyspnea	4	1	1	0	1	4	1	0
Chest pain	0	1	0	0	0	0	1	0
Laryngeal hemorrhage	1	0	0	0	0	0	0	0
Peripheral sensory neuropathy	0	1	0	0	0	0	0	0
Cough	1	0	0	0	0	0	0	0
Productive cough	1	0	0	0	0	0	0	0
Pulmonary fibrosis	0	0	0	0	1	1	2	0
Rib fracture	0	0	0	0	1	0	0	0
Pleural effusion	0	0	0	0	0	1	0	0
Gastro-esophageal reflux	0	0	0	0	0	1	0	0
Soft tissue necrosis	0	0	0	0	0	1	0	0

The number is the number of case with the toxicity described

^a^Within the first 3 months after re-irradiation

^b^ After 3 month after re-irradiaton

**Table 6:** Correlations of Local Control and index of survivals with variables

	LC	PFS	DSS	OS
^a^Histology	0,6	0,3	0,3	0,3
^b^Status of patients	0,7	0,5	0,3	0,3
^c^Number of fractions	0,05	0,09	0,06	0,06
^d^Median EQD2 (10 GY) of re-irradiation	0,4	0,4	0,7	0,7
^e^Median BED2 (10) of re-irradiation	0,4	0,4	0,7	0,7
^f^Median EQD2 (10 GY) cumulative	0,03	0,03	0,1	0,1
^g^Median BED (10) cumulative	0,02	0,05	0,08	0,08
^h^CHT after re-irradiation	0,2	0,09	0,8	0,8
^i^Median time between both treatments	0,4	0,5	0,6	0,6
^j^Median time of first progression of the disease	0,6	0,5	0,3	0,3

^a^ Histology: Adc(adenocarcinoma), Scc (Squamous cell carcinoma), Sclc (Small cell lung cancer), Pet+, Without Histology

^b^ Single Lesion vs. Multiple Lesions

^c^ Single Fraction vs. Multiple Fractions

^d^ EQD2(10) ≥83 Gy vs. < 83 Gy

^e^ BED(10) ≥100 Gy vs. < 100 Gy

^f^ Cumulative EQD2(10) ≥167 Gy vs. < 167 Gy

^g^ Cumulative BED (10) ≥200 Gy vs. < 200 Gy

^h^ CHT(Chemotherapy) after re-irradiatio: Yes vs. No

^i^ Median time between both treatments ≥18 months Vs. < 18 months

^j^ Median time of the first progression of disease ≥10 months vs. < 10 months

### Local control

**Fig. 1:**
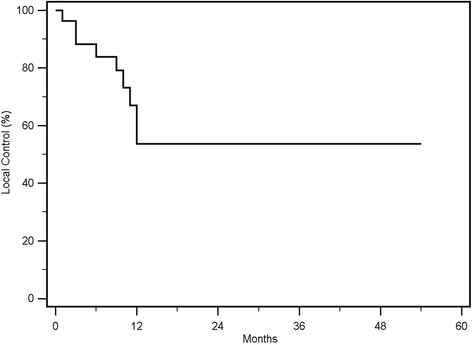
Kaplan Meier curve showing Local Control

**Fig. 2:**
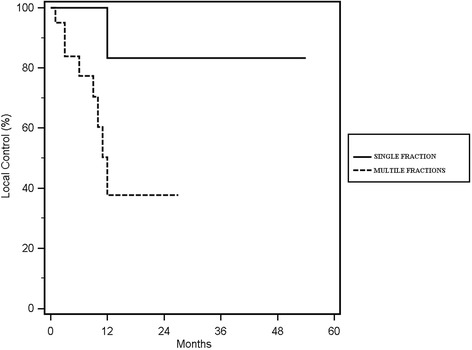
Kaplan Meier curve showing Local Control according with the number of fractions

**Fig. 3:**
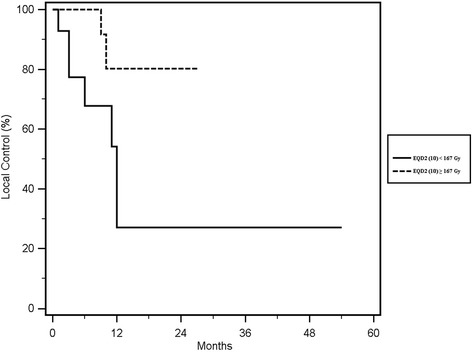
Kaplan Meier curve of Local Control according to the sum of median EQD2 (10) 167 ≥ Gy vs < 167 Gy

**Fig. 4:**
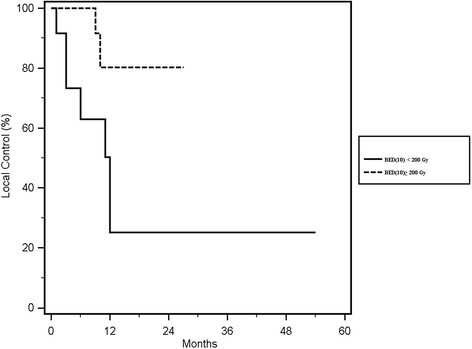
Kaplan Meier curve of Local Control according to the sum of median BED (10) 200 ≥ Gy vs < 200 Gy

Local control was reached in 18 out of 27 (66%) re-irradiated lesions, with rates of 67 and 54% at 1- year and 2- years respectively (Fig. 1). In the univariate analysis of the single fraction vs. multiple fractionation, the median cumulative EQD2(10) of the two treatments ≥167 Gy vs. < 167 Gy and the median cumulative BED(10) of the two treatments, ≥ 200 Gy vs. < 200 Gy showed a statistically significant correlation with local control, respectively with a *p* value of 0.05 (Fig. 2), 0.03 (Fig. 3) and 0.02 (Fig. 4). No other statistically significant correlations were found for the other variables. Considering the heterogeneity of patients was not relevant to consider the other survival index as PFS, DSS and OS.

### Toxicity

The treatment was well tolerated. The maximum recorded toxicity was Grade 3. For 16 lesions (59%) treated we did not find acute toxicity and also for 16 lesions (59%) we did not register late toxicity. Acute and late toxicity are summarized in Table 5. There was one case of dyspnea (4%) G3 as acute toxicity. The same patient (4%) had dyspnea and fibrosis G3 associated with rib fracture G1 as late toxicity. The lesion was in-field. The dose was 50 Gy in 5 fractions, the BED (10) sum was 232.5 Gy, the EQD2(10) sum was 193.8 Gy and the volume was 27 cm^3^. There was one case (4%) of chest pain G3 as late toxicity. It was an in-field lesion. The dose was 45 Gy in 3 fractions, the BED (10) sum was 232.5 Gy, the EQD2 (10) sum was 193.8 Gy, and the volume was 11 cm^3^. There was one case (4%) with pleural effusion G2 associated with pulmonary fibrosis G3, as late toxicity. It was an out-field lesion. The dose was 50 Gy in 5 fractions, the BED (10) sum was 172 Gy, the EQD2(10) sum was 148.3 Gy. The volume was 46.6cm^3^. All the lesions had a peripheral localization.

## Discussion

Primary and metastatic lung diseases often relapse, creating difficulties in the type of therapeutic approach to be use. The relapses may occur in different sites: in the same site or another site of lung or in the lymph node. It can relapse outside of lungs. Generally the first approach for lung disease recurrence is surgery or chemotherapy. Actually progression in technologies, especially SBRT, gives a new treatment approach, not only as palliative intent [9, 10]. There are few data about the efficacy, the doses, the number of fraction and the ideal time between the two treatments. Usually, the target of the first treatment is larger than the target of retreatment. More attention is focused about toxicity, involving more reasonable choice of doses and volume of retreated lesions [11]. The aim of this study was to evaluate the efficacy and tolerability of SBRT re-irradiations treatment of curative intent. We had a LC of 66%, with rates of 67% at 1- year and 54% at 2- years. The others studies showed rates of LC from 59 to 95% at 1- year and from 50 to 92% at 2- years [12]. Liu et al. at MD Anderson in a retrospective study have analysed 386 patients with primitive or metastatic lung disease treated by SBRT for a single lung recurrence, with a maximum diameter ≤ 4 cm. The dose was 50 Gy in 4 fractions, the local control was 95% at 1-year, the median follow-up was 16 months [13]. Kilburn et al. studied a group of 33 patients with primitive and metastatic lung disease treated by SBRT for in-field and out-field lesions. The dose was 50 Gy in 10 fractions. The local control at 2 years was 67%. The median follow-up was 17 months. They found greater efficacy in terms of local control with multiple fractions vs. single fractionation (Log Rank, *p* = 0.006) [14]. As know, the tumor radio resistance depends on the degree of hypoxia. Therefore, if we have more fractions we have more time to reduce the degree of hypoxia and improve the tumor response [12]. In our study, we observed a statistically significant correlation of single fraction vs. multiple fractions (*p* = 0.05), median cumulative EQD2(10) of the two treatments ≥167 Gy vs. < 167 Gy (*p* = 0.03) and median cumulative BED (10) of the two treatments ≥200 Gy vs. < 200 Gy (*p* = 0.02). Comparing these data with those of other studies, we observed discordance about local control results and number of fraction. A possible reason is that the lesions treated with single fraction are smaller than lesions treated with multiple fractions. We did not find any statistical correlation with volume of lesions. Regarding the dose, in terms of BED(10) and EQD2(10), our data confirm the data of Reyngold et al. They found a statistical correlation between LC and BED(10) ≥ 100Gy for the re-irradiation (*p* = 0.04) [4]. Trakul et al. showed good results on correlation between LC and BED when the time between the two treatments was greater than 16 months (*p* = 0.042) [15]. Reyngold et al. confirmed these good data with an interval greater than 36 months (HR = 0.25, p = 0.05). In addition, the same study showed good results on LC for a PTV volume < 75 cm^3^ (*p* = 0.03) and a KPS ≥ 80 (p = 0.03) [4]. Kilburn et al. found a correlation between PTV and LC (p = 0.03) [14]. The lesion volume is very important to define the radio resistance, because in a bigger volume there were a lot of hypoxic cells [7]. We did not find the same correlations in our study. In our study no major toxicity findings or fatal events were identified. The tolerance was good, with 59% of cases with no acute toxicity and the same percentage with no late toxicity. The maximum acute toxicity was one case of dyspnea G3. The maximum late toxicities were a case of dyspnea G3, a case of chest pain G3 and two case of pulmonary fibrosis G3. There were no correlation with dose, localization and volume of lesions. Kelly et al. showed a correlation between out-field lesions and Grade 3 pneumonitis (p = 0.03). However, they did not find correlation with volume, lesion localization (central or peripheral), dose or with history of lung disease. Patients treated for in-field lesions had more frequently chest wall pain and low rates of pneumonitis, while patients treated for out-field lesions had a higher frequency of pneumonitis and less probability of chest pain. Radiation pneumonitis is an inflammatory process. One hypothesis is that previously irradiated areas can be fibrotic, and less susceptible to the inflammatory process caused by a new radiotherapy. No clear explanation can be found for chest pain. One hypothesis is that the nervous damage, fractures and myositis were caused by the re- irradiation [16]. Trovò et al. found an association between the dose received from the heart and the incidence of radiation pneumonitis. Patients who developed grade 3 to 5 pneumonitis had a higher heart Dmax (mean Dmax = 27 Gy), *p* < 0.05 [17]. Liu et al. described pre-SBRT parameters that could influence the incidence of Grade 3–5: ECOG PS 2–3 (*p* = 0.008), a pre-SABR FEV1 ≤ 65% (*p* = 0.012), a V20 ≥ 30% in the composite plan (*p* = 0.020), and previous bilateral mediastinal PTV (*p* = 0.024) [13]. The limits of our study were a small number of patients, a lack of homogeneity in the first treatment and re-irradiation, and a missing algorithm able to sum the doses for OARs and target between the two treatments. We have not reported the results about the index of survival, the correlation with variables because patients and tumor characteristics were too heterogeneous and the study sample was too small to find statistically significant results.

## Conclusions

In this study we showed that SBRT may represent a new chance of treatment of curative intent in the recurrence lung disease. Toxicity was acceptable. However, more knowledge and studies with a higher number of patients, and greater homogeneity of treatment and longer follow-up could help to understand better who is the best candidate to obtain optimal results in terms of LC and toxicity.

## Abbreviations

18FDG PET-CT: PET 18-F-fluorodeoxyglucose (FDG): Positron EmissionTomography integrated with Computed Tomography
3D: 3 Dimensional
4DCT: 4Dimensional Computed Tomography
BED (10): Biological Effective Dose (α/β = 10 Gy)
cc: Centimeter Cubic
CI: Confidence Interval
CRT: Chemo-Radio-Therapy
CTCAE: Common Terminology Criteria for Adverse Events
Dmax: Dose Maximum
DSS: Disease Specific Survival
DVH: Dose Volume Histogram
ECOG: Eastern Cooperative Oncology Group
EQD2 (10): Equivalent Dose in 2 Gy (α/β = 10 Gy)
FEV1: Forced Expiratory Volume in one second
G: Grade
GTV: Gross Tumor Volume
HR: Hazard Ratio
ITV: Internal Target Volume
KPS: Karnofsky Performance Status Scale
LC: Local Control
MIP: Maximum Intensity Projection
MV: Mega Volt
NSCLC: Non-Small Cell Lung cancer
OARs: Organs At Risk
OS: Overall Survival
PFS: Progression Free Survival
PS: Performance Status
PTV: Planning Tumor Volume
RECIST: Solid Tumors Response Evaluation Criteria
SBRT: Stereotactic Body Radiation Therapy
TBCT: Total Body Computed Tomography
V20 ≥ 30%: Percent volume of lung exposed to at least 20Gy ≥30%
